# Specialty preferences and influencing factors: a repeated cross-sectional survey of first- to sixth-year medical students in Jena, Germany

**DOI:** 10.1186/s12909-018-1200-8

**Published:** 2018-05-09

**Authors:** Diana Grasreiner, Uta Dahmen, Utz Settmacher

**Affiliations:** 1 Klinik für Allgemein-, Viszeral- und Gefäßchirurgie, Experimentelle Transplantationschirurgie, Universitätsklinikum Jena, Drackendorfer Str. 1, 07747 Jena, Germany; 2 Klinik für Allgemein-, Viszeral- und Gefäßchirurgie, Universitätsklinikum Jena, Am Klinikum 1, 07747 Jena, Germany

**Keywords:** Postgraduate specialty, Medical student, Influencing factor, Gender, Germany

## Abstract

**Background:**

Given the expected increase in those entering retirement, the number of practising physicians is predicted to decrease. Conversely, the number of physicians needed is set to increase, due to higher demands resulting from the increasing average age of the German population. This may cause a deficit in the availability and accessibility of medical care for the population in Germany, as well as in other countries.

As such, there needs to be a specific focus on the next generation of physicians. Will they fill the gap in those medical specialties where it is most needed? This study aims to investigate (a) preferences for medical specialties over time and (b) the reasoning behind these preferences among students.

**Methods:**

Over three subsequent years, all medical students from the Jena Faculty of Medicine were repeatedly invited to participate in an online survey. The questionnaire consisted of three parts to explore the students’ (1) preferred postgraduate specialty, (2) the reasons for their decision and (3) socio-demographic data.

Data analysis was performed using Fisher’s exact tests and logistic regression analysis.

**Results:**

The number of students completing the questionnaire in a given year ranged from 180 to 320, resulting in a total number of 720 completed questionnaires. Between 40 and 50% of the students preferred internal medicine as postgraduate specialty. About 25% of the students were interested in a surgical specialty. Diagnostics and psychiatric medical fields were preferred by about 10% of all students for each field in each year of the survey. A large percentage (about 18%) of the students remained undecided. The factors influencing the students’ specialty preferences were most frequently reconciliation of work and family life, career goals as well as predicted workload. The factors depended on the preferred medical specialty.

**Conclusion:**

The influencing factors should be taken into account for recruiting prospective residents. Doing so could increase the chance to attract the number of physicians needed to ensure adequate medical care in the field of interest, according to the growing health needs of the population.

## Background

The number of practising physicians in Germany is likely to decrease, due to the expected increase in the average age of the German population [1–3]. In addition, the physicians themselves are ageing, and a large proportion of them will retire soon [1, 4–6]. Furthermore, according to the Association of German Surgeons (Bund Deutscher Chirurgen), the rate of students selecting surgery for their postgraduate specialty is declining [7–9].

In contrast, the number of physicians needed is set to increase, due to higher demands from the population in relation to the number and extent of medical treatments [4, 7, 10]. Altogether, this may cause a deficit in the availability and accessibility of healthcare for the population in Germany and other countries. In the UK, the Netherlands, France and Switzerland, as well as non-European countries, such as Canada, Brazil and Saudi Arabia, several surveys have been conducted to explore reasons influencing students in their specialty selection [11–18]. In recent years, work-life balance, self-fulfilment and income have often been cited as decision-making factors in these publications.

However, the factors influencing the selection of postgraduate medical specialties among German medical students are not well understood [7].

This study aims to investigate (a) the preference for medical specialties over time at the Jena Faculty of Medicine and (b) the reasons for selecting surgery or other medical specialties. In our study, we took a closer look at surgical specialties due to the deteriorating situation in this medical field, which is due, e.g., to difficult working time arrangements and a large proportion of retiring surgeons in Germany [8].

This knowledge could help to improve the recruitment of future physicians in Germany; without an awareness of the influencing factors, they cannot be taken into account when seeking to attract new residents [19, 20]. Therefore, it is essential to find out if there are any influences from changes in specialty preference over time or gender-related differences.

The study was performed in 2014, 2015 and 2016 at the Jena Faculty of Medicine, when a new curriculum was being implemented. This change was intended to increase the level of practical experience for each student with a special focus on the ambulatory, clinical or research sector. The so-called JENOS project included theoretical and practical teaching and ended with an objective structured clinical examination (OSCE) [21].

## Methods

### Study design and distribution

To answer these questions, we designed a repeated cross-sectional survey. In turn, we invited medical students from the first- to the sixth-year at the Medical Faculty of Jena in three consecutive years. The students were asked to complete an online questionnaire during a six-week period in the summer semester of 2014, 2015 and 2016 [22].

The survey was generated using SoSciSurvey and mainly distributed electronically via email distributors and additionally through students’ Facebook groups according to their year of study. Students registered with the main email distributor for medicine, as well as the email distributor according to their year of study, received two electronic invitations. A reminder was sent after 3 weeks via Facebook.

A pretest was performed to ensure that the items were understandable and unambiguous. Randomly selected students examined the survey, and the questionnaire was revised according to their comments and suggestions. As a result, we adapted the sequence of the questions of the survey.

### Questionnaire

A three-part questionnaire was developed on the basis of a literature review [19, 22, 23]. The first part of the survey asked the students which postgraduate specialty they preferred from a list of 32 possibilities. For statistical analysis purposes, the selected specialties were aggregated into five large groups (internal medicine, surgery, diagnostics, psychiatry and undecided) [Table 1] [24].

**Table 1:** Summary of the five groups of specialties

Internal medicine	General medicine
	Anaesthesiology
	Paediatrics and Youth Medicine
	Neurology
	Cardiology
	Internal medicine
	Haematology and Oncology
	Physical and Rehabilitation medicine
	Endocrinology and Diabetology
	Gastroenterology
	Nephrology
	Pneumology
	Rheumatology
	Dermatology and Venerology
	Occupational medicine, Public health, Hygiene and Environmental medicine
	Angiology
	Pharmacology
	Clinical Pharmacology
	Pharmacology and Toxicology
	Radiotherapy
Surgery	Orthopaedics and Trauma surgery
	Gynaecology and Obstetrics
	Ophthalmology
	Heart surgery
	Neurosurgery
	Abdominal surgery
	General surgery
	Oral Maxillofacial surgery
	Vascular surgery
	Paediatric surgery
	Otorhinolaryngology
	Urology
	Thoracic surgery
	Plastic and Aesthetic surgery
Diagnostics	Radiology and Nuclear medicine
	Forensic medicine
	Laboratory medicine, Human genetics, Transfusion medicine
	Microbiology, Virology, Infection epidemiology
	Pathology, Neuropathology
Psychiatry	Psychiatry and Psychotherapy
	Psychosomatic Medicine and Psychotherapy
	Childhood and Adolescent Psychiatry and Psychotherapy
Undecided	I’m still undecided

Overview of the medical specialties and to which larger group they belong in our survey

In the second part, we explored the reasons underlying their specialty preferences. The participants were asked to select which influencing factors they regarded as important when selecting their specialty. The predefined factors covered characteristics of medical specialties and the personal preferences of the students in terms of their future working life.

In the third part of the survey, we asked for socio-demographic data, such as age, gender and marital status.

Depending on the question, the students’ responses were collected using Likert scales, yes/no answers, options menus or free input fields. For each question, two alternatives were available to choose from: “I don’t know” or “I don’t want to answer this question”.

No monetary or other incentive was offered to the participants.

### Statistical analysis

Results were expressed as both absolute numbers and percentages. The response rate was determined by calculating the ratio between the number of completely filled questionnaires and the number of invited participants.

Fisher’s exact tests were used to determine whether there were any differences (H_1_) between female and male students concerning the preferred medical specialties or not (H_0_) [Table 2].

**Table 2:**
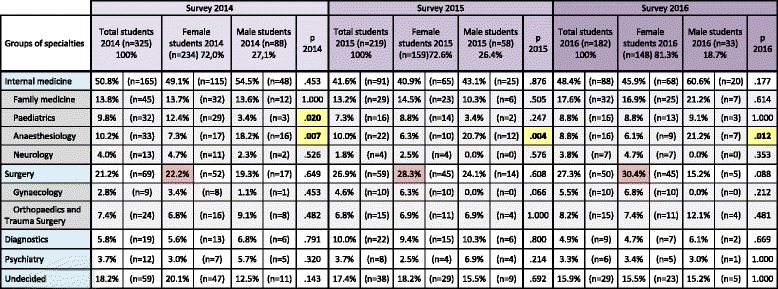
Specialty distribution over a period of three consecutive years [29]

Percentages and numbers respectively relate to each year. White fields: Data for the five large groups of medical specialties. Grey fields: Data for some individual medical specialties. Yellow fields: The *p*-values of individual medical specialties with large differences in the specialty preference between female and male students. Purple fields: Percentages of female students preferring surgery

Logistic regression analysis was conducted to investigate the joint effect of the influencing factors. We used the preference for a group of specialties as dependent variable in the regression model, e.g., the students preferred “internal medicine” or they did not prefer this as specialty (yes or no). The explanatory or independent variables of the multivariable analysis were influencing factors, such as “workload”, “income” or “reconciliation of work and family life” [Table 3]. In Table 3, the separate logistic regression analyses for the five groups of specialties are shown. In turn, we performed a regression analysis for each of the large groups of specialties, one by one.

**Table 3:**
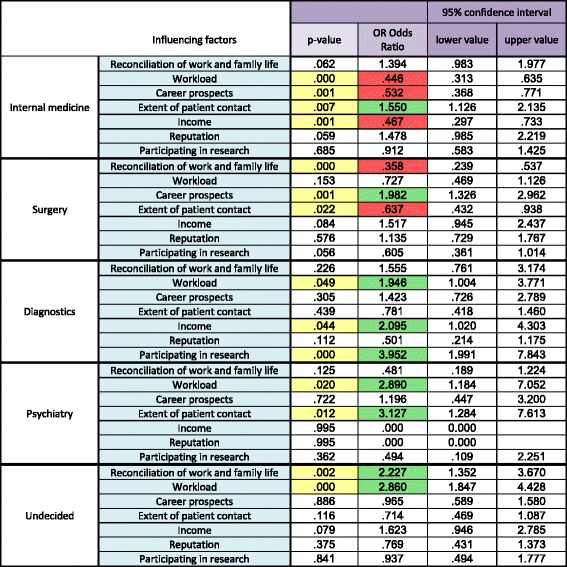
Factors for specialty selection

Method: The students were asked to decide whether any of the given factors influenced their decision to select a given specialty, and to rate each factor separately. Green cells: Positive attribution. Red cells: Negative attribution. Yellow cells: significant *p*-values

The odds ratio (OR), as determined by the logistic regression analysis, was considered as an estimate of the relative risk concerning which factors had and did not have an influence on the students’ specialty preference [25]. An indication of the increased or decreased odds was given by the association with the influencing factors (green or red markings in [Table 3]). An OR above the value of 1 was considered as a (positive) influencing factor and an OR below 1 as a factor with a negative or no influence on the students’ decision-making process.

The OR is simply the ratio between the following two ratios: The ratio between “the student preferred a particular specialty” and “the student did not prefer a particular specialty”, and the ratio between “a positive correlation with an influencing factor” and “a negative correlation with an influencing factor” [26].

A *p-value* < 0.05 was considered as statistically significant. Statistical data analysis was performed using SPSS version 23 (IBM Corp., Armonk, NY, USA) and Office 2016 version of Excel (Microsoft, Redmond, WA, USA).

### Ethical approval

According to the Ethics Committee of the Jena Faculty of Medicine, formal ethical approval was not needed since anonymity of the participating students and data safety were ensured; nor did the study involve any patient data.

## Results

### Response rate and socio-demographic data

The response rates ranged between 9.2% and 18.5% of all medical students in the given year, as shown in Table 4. In total, 720 questionnaires were completed.

**Table 4:** Socio-demographic characteristics

		Survey 2014		Survey 2015			Survey 2016		
	Participating students in Jena 2014 (*n* = 322) Response rate 18.5%	All medical students in Jena 2014 (*n* = 1740)	German medical students 2014 (*n* = 87,863)	Participating students in Jena 2015 (*n* = 217) Response rate 12.2%	All medical students in Jena 2015 (*n* = 1782)	German medical students 2015 (*n* = 89,998)	Participating students in Jena 2016 (*n* = 181) Response rate 9.2%	All medical students in Jena 2016 (*n* = 1971)	German medical students 2016 (*n* = 91,938)
Gender, *n* (%)									
Female	234 (72.0%)	1167 (67.1%)	53,352 (61%)	159 (72.6%)	1188 (66.7%)	54,638 (61%)	148 (81.3%)	1310 (66.5%)	56,240 (61.2%)
Male	88 (27.1%)	573 (32.9%)	34,511 (39%)	58 (26.5%)	594 (33.3%)	35,360 (39%)	33 (18.1%)	661 (33.5%)	35,698 (38.8%)
Not specified	3 (0.9%)			2 (0.9%)			1 (0.5%)		
Age, median	24.0	24.8	not available	24.0	24.9	not available	24.0	24.5	not available
Civil status									
Single	110 (33.8%)	not available	not available	76 (34.7%)	not available	not available	73 (40.1%)	not available	not available
In a relationship	179 (55.1%)			112 (51.1%)			86 (47.3%)		
Married	24 (7.4%)			12 (5.5%)			17 (9.3%)		
Not specified	12 (3.7%)			18 (8.2%)			6 (3.3%)		
Children									
Yes	24 (7.4%)	not available	not available	15 (10.5%)	not available	not available	15 (8.2%)	not available	not available
No	295 (90.8%)			196 (89.5%)			164 (90.1%)		
Not specified	6 (1.8%)			8 (3.7%)			3 (1.6%)		

The sample population was almost similar to the entire medical student population in Jena and in Germany, in terms of gender and age. Demographic characteristics of the respondents, in comparison to all German medical students, are shown in Table 4. The median age of the participants was 24 years, in the range 18–44 years, compared to a median age of 24.9 years of the total population of medical students in Jena.

The distribution of socio-demographic data changed slightly over all 3 years. Gender distribution of the participants was unequally distributed, as it is nowadays throughout the whole population of medical students in Germany. Nearly three quarters of the respondents in all 3 years were female. The number of female participants was slightly higher compared to the total population of female medical students from the Jena Faculty of Medicine. Compared to the total population of female medical students in Germany, females in Jena were somewhat over-represented.

### Preferred medical fields - general distribution

**Fig. 1:**
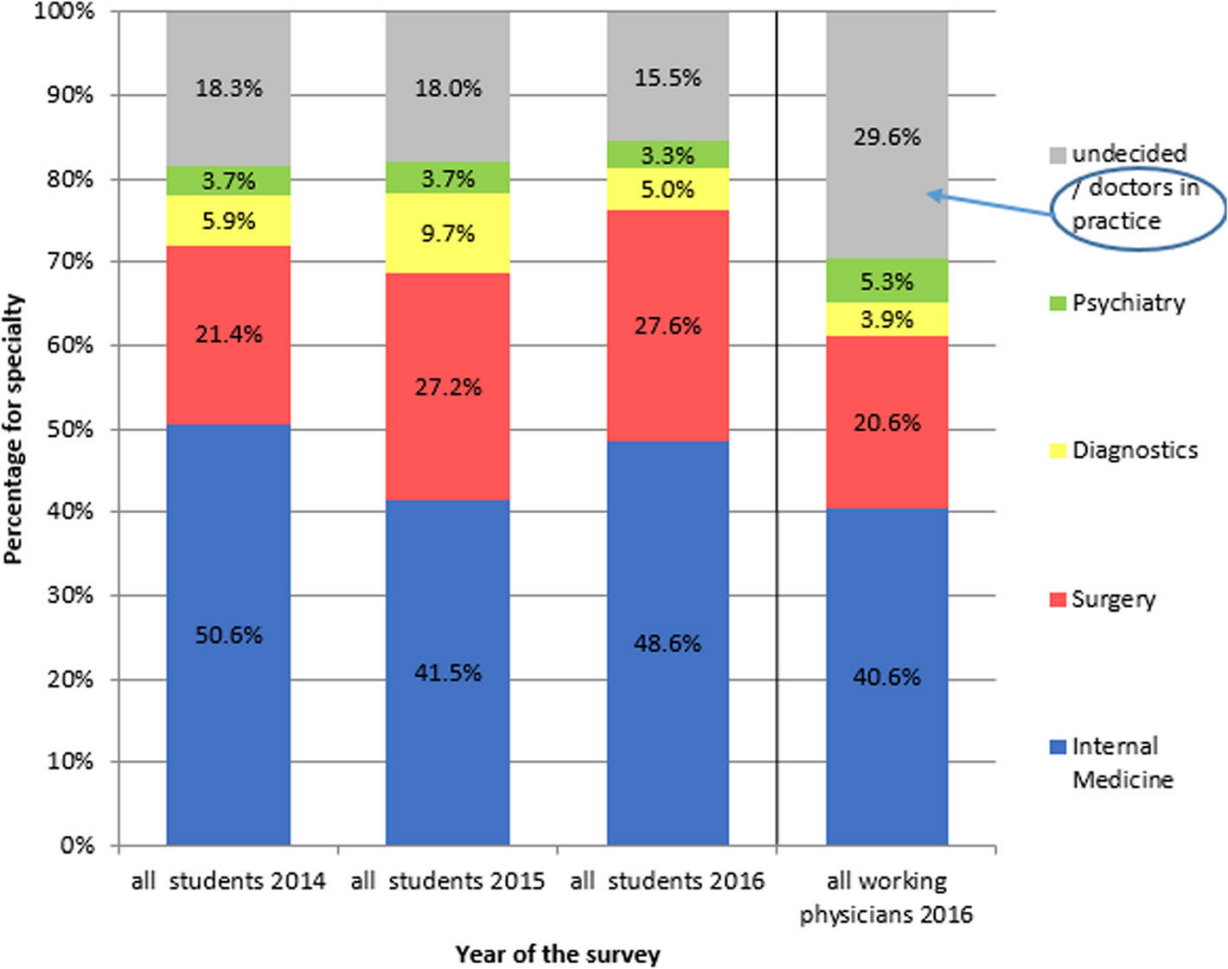
Specialty selection from 2014 to 2016. Distribution of preferences for medical specialty according to gender and study year [31]

Separate analysis of the results from the years 2014, 2015 and 2016 revealed a stable distribution of the preferred medical fields between female and male students [Table 2]. It emerged that about half of the participants were interested in internal medicine, a quarter in surgical fields and about 10% in diagnostics or psychiatric fields [Fig. 1, Table 2]. A high proportion, i.e., nearly one fifth, of the students were undecided about their future postgraduate specialty. These students could potentially be attracted to under-represented medical fields, if their reasons influencing their decision-making were better understood.

Gender-related differences were found in Anaesthesiology and Paediatrics. The former specialty was preferred more by male students in all 3 years, whereas the latter was preferred more by female students (but only in 2014).

The students preferring surgery were increasingly female, which is in accordance with the findings of the German Medical Statistics Department. This situation could lead to an increasing shortage of surgeons due to family-related employment breaks, although flexible working models could help to address this.

### Factors influencing the preference of a given medical specialty

**Fig. 2:**
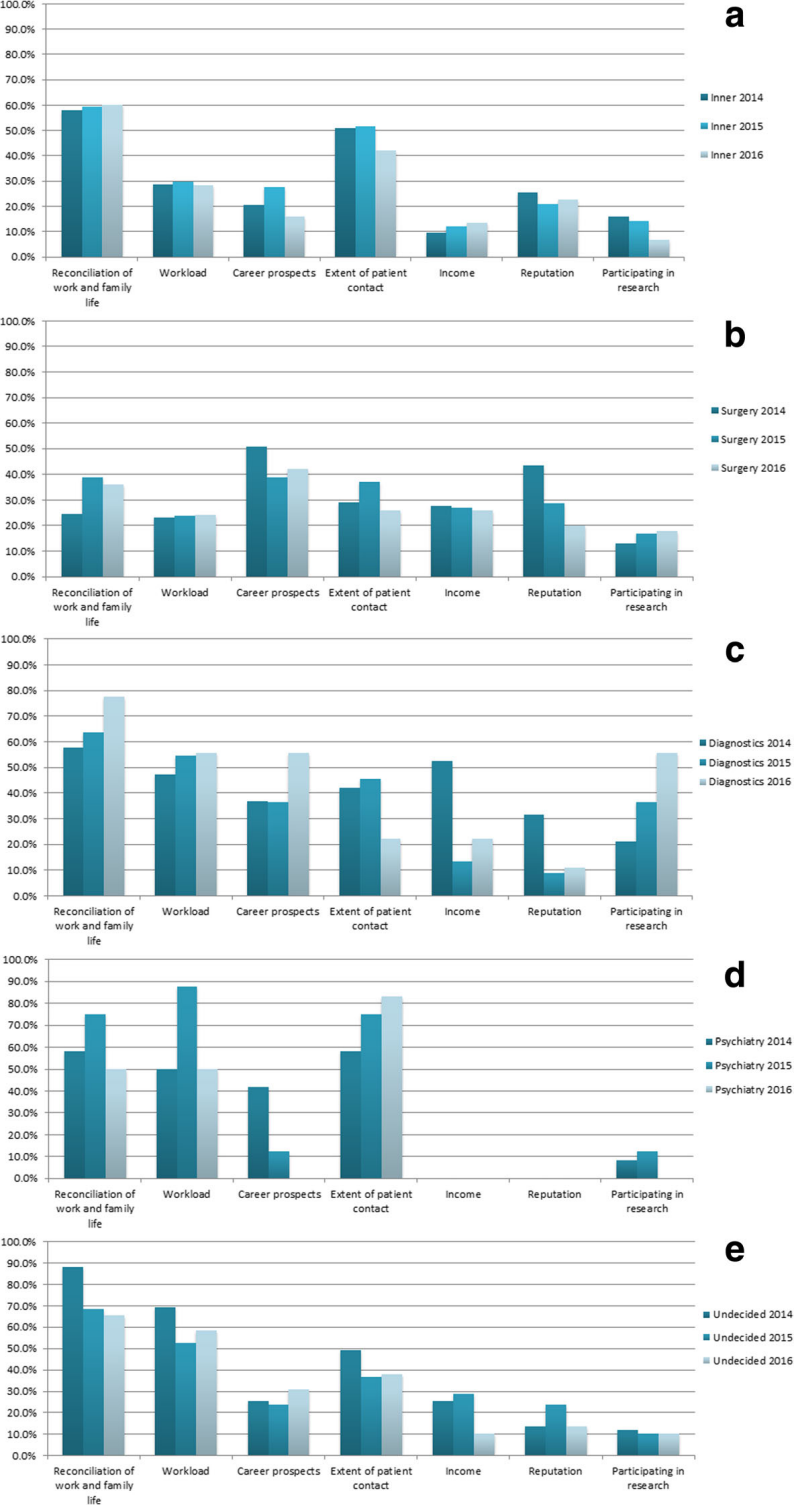
Influencing factors for 3 years. Distribution of the influencing factors for the five groups of medical specialties for three subsequent years. **a** Internal medicine. **b** Surgery. **c** Diagnostics. **d** Psychiatry. **e** Undecided students

Besides “reconciliation of work and family life”, the factors “workload” and “career prospects” were indicated most frequently as influences on the preference regarding the postgraduate specialty, as shown in Table 3 and in Fig. 2a-e. The reason for preferring one of the specialties in the five main medical groups differed considerably, depending on the selected group.

#### Internal medicine

Students preferring internal medicine had a strong positive association with the “extent of patient contact” (OR 1.550) [Fig. 2a, Table 3]. In contrast, they were less influenced by career-related factors such as “workload” (OR 0.446), “career prospects” (OR 0.532) or “income” (OR 0.467) compared to students preferring other specialties.

#### Surgery

Career-associated factors, such as “career prospects” (OR 1.982), were listed by students selecting surgery as strongly influencing their preference [Fig. 2b, Table 3]. In contrast, students preferring other specialties less often rated career-related issues as important in their decision-making.

During all 3 years of the survey, students preferring surgery attached less importance to “reconciliation of work and family life” (OR .358) or to the likely “extent of patient contact” (OR .637) compared to students preferring non-surgical specialties [Table 3].

#### Diagnostics

It should be emphasized that the students preferring diagnostic specialties rated the possible “participation in research” (OR 3.952) as a strong influencing factor in their decision-making [Fig. 2c, Table 3]. This was not the case with other medical specialties. A large impact was observed in relation to the students’ future “income” (OR 2.095), as well as prospective “workload” (OR 1.946).

#### Psychiatry

The students who preferred psychiatric specialties acknowledged the “extent of patient contact” (OR 3.127) [Fig. 2d, Table 3] as a strong influencing factor. In almost the same manner, the students reported a strong positive association with their prospective “workload” (OR 2.890).

#### Undecided students

The group of undecided students is of special interest. The majority of these students indicated that “reconciliation of work and family life” (OR 2.227) and the expected future “workload” (2.860) were factors of great importance for them, despite having no preferred specialty at the time of the survey [Fig. 2e, Table 3].

The influencing factors of undecided students were in contrast to those of students preferring, for example, surgical medical fields or internal medicine [Table 3].

## Discussion

The present single centre study investigated the preferences among medical students concerning the selection of a postgraduate specialty and their reasons behind this process.

It should be noted that, according to our study, the interest in surgery does not decline during the course of studies, as reported previously by Ansorg et al. [7] and Paulmann et al. [27], but remains constant. Ansorg et al. and Paulmann et al. observed a declining interest in surgical fields, or rather a lower proportion of female students interested in surgical fields. In contrast, Diderichsen et al. [28] reported an unchanged interest, which is in accordance with our results. This is further supported by the national statistics regarding the distribution of board-certified physicians [29–31]. According to the German Federal Statistical Office, the rate of surgeons remained stable at 20% over the last 16 years [31–33].

### Specialty preferences and comparison with working physicians

According to our results, the distribution of preferred postgraduate specialties among Jena medical students is comparable to the current distribution of specialties among currently working physicians in Germany [Fig. 1]. [29–33]. Selected specialties of the study population were compared to statistics for German physicians [14]. The distribution of medical specialties among board-certified working physicians and the preferences of the students was very similar, suggesting an unchanged interest in the different fields of medicine [see also Table 5] [29–33]. This corresponds to the findings of Diderichsen et al. [28], who stated, in their cross-sectional study, that Swedish medical students show nearly the same preferences in their specialty selection, irrespective of their gender. The proportion of students selecting surgery in Jena increased slightly from 21.4% in 2014 to 27.6% in 2016. If our results hold true, the potential shortage of surgeons would be limited to what is required in order to meet the growing demand from a population with increasing age and healthcare needs. As female students outnumbered male students, the shortage in surgical residents due to family-related absences requires a rethink regarding working time models in understaffed medical fields [4, 7].

**Table 5:**
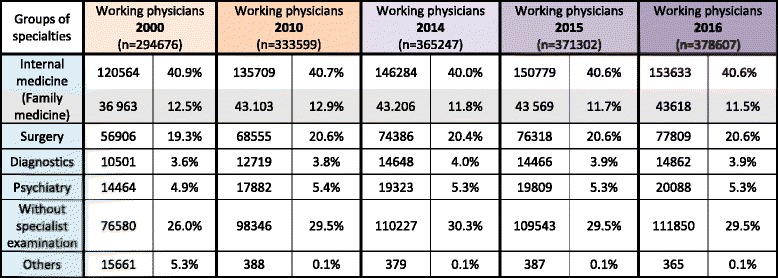
Summary of working physicians

Chronological sequence of working physicians highlighting the steady increase in the number of female surgeons. White fields: Data for the five large groups of medical specialties. Grey fields: Data for an individual medical specialty

The group of undecided students is of particular interest, because of the possible recruitment of these students in medical specialties with a shortage of residents. According to Al-Fouzan et al. [22], the proportion of undecided students could be diminished with the help of formal career counselling during medical school. This could be implemented according to the Canadian guidelines [34]. An optimal time to perform career counselling is the end of the clinical part of studies, as well as the beginning of the residency, which could be carried out as individual counselling or as a group event, especially for disadvantaged students [35]. In addition, there may be a lack of role models in some understaffed specialties [5, 36]. Role models are characterized by their expertise and, in particular, their approachability [36].

In our study, the reasons rated as important by the majority of undecided students were different to the reasons given by students primarily interested in surgery. Consequently, the factors “workload” and “reconciliation of work and family life” should be taken into account when trying to attract currently undecided students into potentially under-represented specialties, such as surgery. In other words, special effort should be focused on offering suitable working conditions to this cohort of students.

### Factors influencing specialty selection

We identified the factors influencing the selection of postgraduate medical specialties including personal motivations, career-associated reasons and work-life balance. Personal aspects of future life planning and characteristics attributed to a given specialty had a large influence on the selection of the postgraduate specialty.

Characteristics commonly attributed to surgery include promising career prospects, high workload and good reputation. Kiolbassa et al. [19] also stated that students selecting surgery are more concerned about their career prospects and their reputation than students selecting other disciplines. These characteristics were also the key influencing factors among students selecting surgery as their postgraduate specialty in our study, as well as in the survey conducted by Khader et al. and other studies [18, 19, 28, 37–41]. According to Khader et al. [37], in the main, male students preferring surgery were highly influenced by factors such as prestige and income, whereas females did not rate these factors as being of the utmost importance.

In contrast, Kaderli et al. [42] and others [5, 19, 43] stated that factors such as work-life balance and family planning do not equate with seeking a undemanding lifestyle, but with having the time to fulfil life goals besides work [42]. Consequently, surgery is less often selected by students who attach importance to family life, as a complement to their working life. These students tend to select internal medicine, diagnostics or psychiatry as their preference. Similar results have also been reported in studies by Alers et al., Takeda et al. and others [18, 19, 44–47]. According to Alers et al. [45], Diderichsen et al. [28] and Harries et al. [48], female physicians tend to work part-time more than their male colleagues. These women often select disciplines such as general medicine or internal medicine because they associate them with family friendliness [28, 45]. Takeda et al. [46] and Correia Lima de Souza et al. [15] stated that surgical specialties (surgery, neurosurgery) were associated with having little time for the fulfilment of life goals, whereas other specialties such as ophthalmology, radiology or dermatology allowed time for personal goals. In particular, more female students would prefer to work part-time compared with male students [28, 48]. Consequently, female students follow a different reasoning process in the selection of a specific specialty than male students. Furthermore, the feminisation of medicine, and in particular of surgery, would require a change in thinking about work-life balance, modern working time models, and the participation of females in leadership and research [49].

In summary, the preferences of the medical students in our study almost reflect the distribution of future physicians across Germany [Table 2] [1]. Therefore, the influencing factors for the decision-making process should be taken into greater consideration when addressing a shortage of physicians in certain specialties [50, 51]. For instance, improved working conditions would be required to accommodate the wish for a sustained work-life balance [27, 31]. This would be essential not only to attract students into under-represented medical fields at the postgraduate specialty stage, but also to help them complete their residency and enable them to pursue a successful career as a physician. However, our results confirm that there is a need for the introduction of more flexible working models, career counselling at an appropriate stage during studies, and good mentoring interventions for residents.

### Strengths

This study was conducted at a single medical school in Germany, but as a repeated cross-sectional study addressing all students repeatedly over 3 years. This design has both strengths and limitations. Including other medical schools could have provided a better representation of the study population. However, our sample population had a similar gender and age distribution as nationally reported, suggesting a satisfactory representation.

## Conclusion

According to our study, the interest in different medical specialties remained stable regarding the gender of the students who participated. The decision-making process was found to be affected by the desire to achieve a work-life balance, allowing for career and family commitments to be reconciled.

Our results suggest that extra effort should be focused on adapting working conditions to ensure that pursuing both goals in life is supported. Doing so could help students who are already interested in under-represented medical fields to realize their professional goal, as well as encourage undecided students to consider this specialty. This, in turn, could increase the possibility of recruiting the number of physicians needed to ensure adequate medical care according to the growing demands of the German population.

## Abbreviations

f: Female
H_0_: Null hypothesis, which means that the proportions of female and male students with the characteristic are equal
H_1_: The proportions of the population (female and male students) are not equal
LTFT: Less than full-time training
m: Male
n: Number
OR: Odds ratio, which is an estimate of the relative risk; it gives an indication of the increased or decreased odds associated with influencing factors
OSCE: Objective structured clinical examination
